# Aging and sex affect soluble alpha klotho levels in bonobos and chimpanzees

**DOI:** 10.1186/s12983-018-0282-9

**Published:** 2018-09-19

**Authors:** V. Behringer, J. M. G. Stevens, T. Deschner, R. Sonnweber, G. Hohmann

**Affiliations:** 1 Department of Primatology, Max Planck Institute for Evolutionary Anthropology, Deutscher Platz 6, 04103 Leipzig, Germany; 2 Antwerp Zoo Centre for Research and Conservation, Royal Zoological Society of Antwerp, K. Astridplein 26, 2018 Antwerp, Belgium; 3 Behavioral Ecology and Ecophysiology, Department of Biology, University of Antwerp, 2610 Wilrijk, Belgium

**Keywords:** *Pan paniscus*, *Pan troglodytes*, Ape, Senescence, Sex-specific, Aging

## Abstract

**Background:**

Throughout life, physiological homeostasis is challenged and the capacity to cope with such challenges declines with increasing age. In many species, sex differences exist in life expectancy. Sex-specific differences have been related to extrinsic factors like mate competition and/or intrinsic proximate mechanisms such as hormonal changes. In humans, an intrinsic factor related to aging is soluble alpha klotho (α-Kl). Both sexes show an age-related decline in α-Kl, but throughout life women have higher levels than men of the same age. Sex differences in α-Kl have been linked to a shorter lifespan, as well as to specific morbidity factors such as atherosclerosis and arteries calcifications. In non-human animals, information on α-Kl levels is rare and restricted to experimental work. Our cross-sectional study is the first on α-Kl levels in two long-lived species: bonobos (*Pan paniscus*) and chimpanzees (*Pan troglodytes*). As in most mammals, female bonobos and chimpanzees have longer life expectancy than males.

**Methods:**

We measured serum α-Kl levels of 140 subjects from 16 zoos with an ELISA to examine if α-Kl levels reflect this difference in life expectancy.

**Results:**

In both species and in both sexes, α-Kl levels declined with age suggesting that this marker has potential for aging studies beyond humans. We also found species-specific differences. Adult female bonobos had higher α-Kl levels than males, a difference that corresponds to the pattern found in humans. In chimpanzees, we found the opposite: males had higher α-Kl levels than females.

**Conclusion:**

We suggest that contrasting sex differences in adult α-Kl levels mirror the dominance relations between females and males of the two *Pan* species; and that this might be related to corresponding sex differences in their exposure to stress. In humans, higher cortisol levels were found to be related to lower α-Kl levels. We conclude that there is great potential for studying aging processes in hominoids, and perhaps also in other non-human primates, by measuring α-Kl levels. To better understand the causes for sex differences in this aging marker, consideration of behavioural parameters such as competition and stress exposure will be required as well as other physiological markers.

**Electronic supplementary material:**

The online version of this article (10.1186/s12983-018-0282-9) contains supplementary material, which is available to authorized users.

## Background

“*Some have suggested that ageing is too complicated for serious scientific study, or that it is like a slow-motion car crash — everything just gets wrecked*” [1].

Aging is defined as the age-related deterioration in physiological functions necessary for survival and fecundity [2–4]. Individuals are exposed to challenges to physiological homeostasis throughout life, but the capacity of the coping mechanisms associated with such challenges declines with age. Therefore, aging is the balance between physiological damage and repair [5].

Bonobos (*Pan paniscus*) and chimpanzees (*Pan troglodytes*) are sister species and share life history traits such as slow maturation, late onset of reproduction [6], and phenotypic traits of aging. For example, aging in bonobos and chimpanzees is associated with an accumulation of bone traumata, decreases in bone minerals and bone mass [7], brain weight loss [8], gradual decrease in cognitive and motoric skills [9], and reproductive senescence in females [10, 11] and males [12]. In wild and captive chimpanzees, males have a shorter life expectancy and a reduced survival probability than females [13–15], perhaps due to higher male mortality rates across all age classes [15–17]. Corresponding data from bonobos are biased towards captive individuals but suggest a similar result: males have a shorter life expectancy than females [14]. In wild bonobos, the mortality rate in adult males seems to be higher than in females. The sex ratio during the infant and juvenile period is nearly the same or biased towards males [18], however, the number of adult females usually exceeds the number of adult males [19–21]. This shift in sex ratio between infancy and adulthood implies that mortality rate is higher in males. While these findings in bonobos and chimpanzees are in line with the male sex-specific bias in mortality, a common phenomenon in many mammalian species, it is in contrast to predictions deriving from the nature of male-male and mate competition in these two species [22–24]. In comparison with chimpanzees, aggressive interactions among male bonobos are moderate and physical aggression is rare [25, 26]. Furthermore, between-group relations can seem more relaxed in bonobos, and it is not uncommon that individuals from different groups may feed in the same food patch and engage in affiliative interactions [27, 28]. In contrast, between-group relations in chimpanzees are characterized by xenophobia and males engage in intense physical aggression during encounters, which often have lethal consequences [29–31]. Given the differences regarding the nature of male-male and between-group competition, the impact of extrinsic factors affecting life expectancy is expected to be stronger in male chimpanzees compared to male bonobos. Accordingly, sex differences in life expectancy should be more pronounced in chimpanzees compared with bonobos.

When trying to explain sex-specific aging patterns, intrinsic markers are of strong interest. Investigating physiological markers that change with age is promising to help explain the sex-specific aging patterns observed in the two *Pan* species. Preliminary evidence for sex differences in physiological aging patterns potentially influencing the life expectancy in bonobos and chimpanzees originate from a study on insulin-like growth factor binding protein 3 (IGFBP-3) [32]. This protein is part of the somatotropic axis, which regulates cell proliferation and apoptosis. In humans, the age-related decline in IGFBP-3 levels has been associated with pathologies, e.g., cardiovascular disease [33–35]. In bonobos and chimpanzees, urinary IGFBP-3 levels decline with age in both sexes but are higher in females than in males. In conjunction with data on life expectancy, we concluded that the sex differences in IGFBP-3 levels indicate that high levels of IGFBP-3 in females promote their longevity [32]. Furthermore, the intense male-male competition in chimpanzees would suggest longer life expectancy in females. Therefore, the observed sex differences in longevity in these hominoid species may be caused by intrinsic physiological factors like hormones and/or by extrinsic factors like male-male competition.

One marker for detecting age-related changes in physiology in humans is soluble alpha klotho (α-Kl). The Klotho gene was originally described in a mutant mouse strain that could not express klotho [36]. The klotho mouse strain developed multiple disorders resembling human aging syndromes including, amongst others, atherosclerosis, calcifications in arteries, muscle wasting, and short life expectancy [36–39]. The function of the Klotho gene was confirmed from transgenic mouse strains that overexpressed the Klotho gene and had prolonged average life expectancy [37]. The effect of the Klotho gene on aging and the emergence of age-related diseases is not restricted to mice but has also been found in humans [40, 41] and rhesus macaques (*Macaca mulatta*) [42].

The human Klotho gene encodes either a transmembrane protein or a secreted form [43]. The secreted form, soluble alpha klotho (α-Kl), functions as an endocrine factor [44, 45]. Soluble α-Kl suppresses aging via inhibition of insulin and insulin-like growth factor 1 (IGF-1) signalling [46–48]. Apart from being part in this regulation, α-Kl is involved in the resistance to oxidative stress in mammals [49]. Oxidative stress is connected to the pathogenesis of various disease conditions and by inducing resistance to oxidative stress, α-Kl protects tissues and cells from oxidative damages [45, 49, 50]. This can lead to enhanced longevity [51]. Other functional pathways of the secreted form of α-Kl are calcium homeostasis in the kidneys [38, 46, 52, 53], phosphaturic effects [39, 54], and anti-inflammatory capacity [55]. Furthermore, higher α-Kl levels in humans are associated with longer life expectancy [56, 57], and lower rates of age-related diseases, e.g., stroke and cardiovascular disease [58]. The age-related decline in α-Kl levels assumed to be a consequence of and not the cause for aging [59]. However, klotho deficiency causes symptoms of aging in many organs and tissues [46].

In animals, observed sex differences in life expectancy have also been linked to extrinsic factors. For instance, the intensity of male-male competition has a strong impact on sex differences in life expectancy, with males in polygynous societies experiencing a reduced lifespan compared to monogamous species [2, 60]. Male-male competition can be intense in polygynous groups; when reproductive activity is biased to older adult males, selection pressure on younger males is reduced and investment in maintenance and longevity is promoted [2]. Specific aging patterns reflect reproductive fitness trade-offs between times when individuals are young and later in life. For example, testosterone of young males is associated with morphological and behavioural traits facilitating intra-sexual competition among males. Thus, high testosterone levels may enhance reproductive success in species in which aggressive mate competition leads to higher reproductive success [61–63], but high testosterone levels increase risk-taking behaviour and immune suppression and consequently are thought to reduce life expectancy of males [64]. However, variation in life expectancy between the sexes might also be caused by factors other than intra-sexual competition e.g., in anthropoid primates, the sex with the greater investment in parenting and care for the offspring (usually the mother) has a longer life expectancy [65].

### Aims of our study

The aims of this study were to assess (a) if bonobos and chimpanzees experience an age-related decline in α-Kl levels, and (b) if α-Kl levels are associated with sex differences in life expectancy. We hypothesized that α-Kl levels would be lower in older individuals in comparison with younger ones, and lower in males compared with females. In case α-Kl levels reflect trajectories of aging that are due to extrinsic factors such as intensity of intra-sexual competition [2], we hypothesized that apart from a general age-related decline, sex differences would be more pronounced in chimpanzees and absent or modest in bonobos.

## Results

### Soluble alpha klotho (α-Kl) levels and aging in bonobos and chimpanzees

**Fig. 1:**
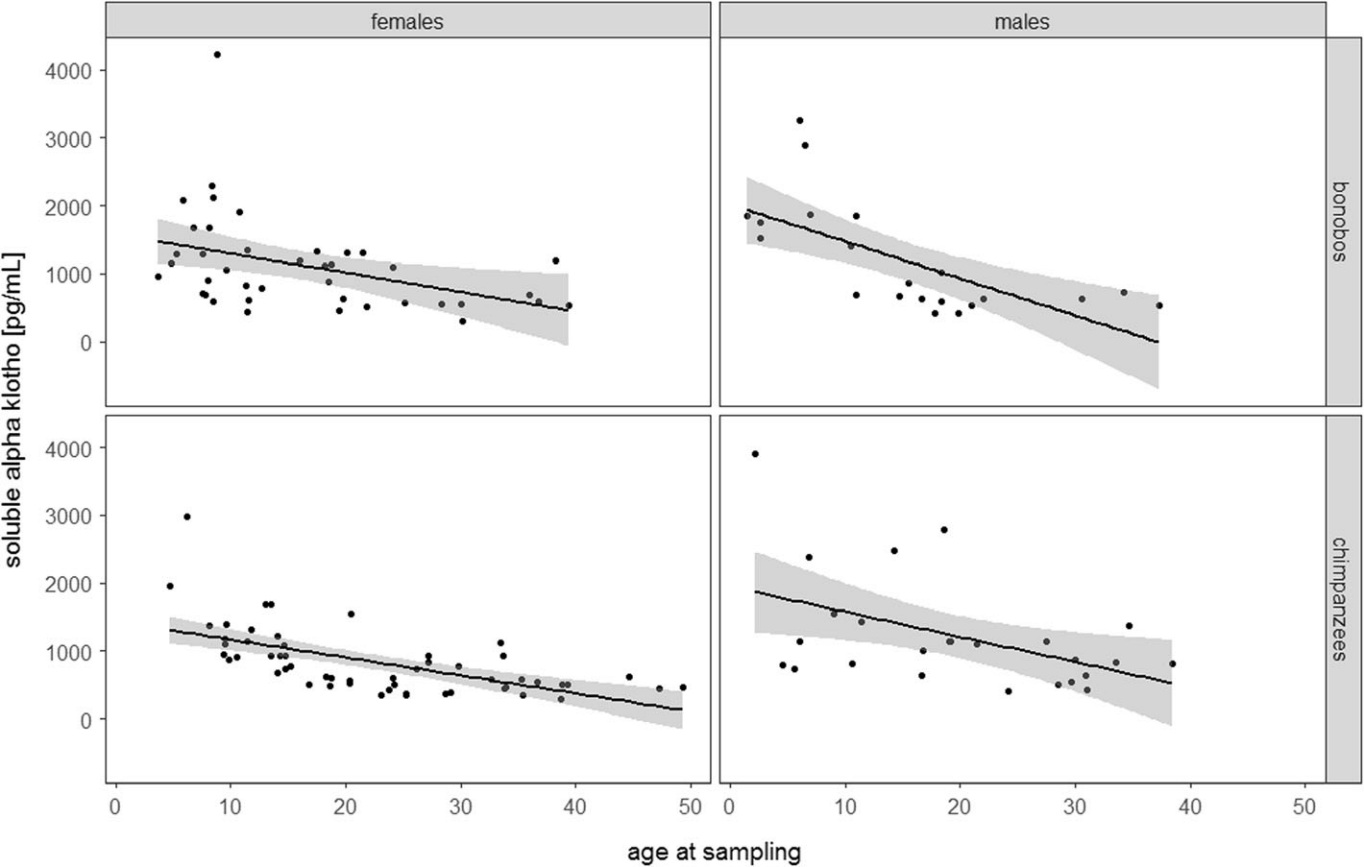
Measures of soluble alpha Klotho levels for females and males of bonobos and chimpanzees in relation to age. Sample sizes: N_bonobos_ = 63 (21 males, 42 females); N_chimpanzees_ = 77 (25 males, 52 females). Shaded areas represent confidence intervals for expected soluble alpha Klotho levels

We ran a linear mixed model (LMM [66]) to examine sex-, species-, and age-specific changes of α-Kl levels throughout life (all-age-model). The all-age full model explains the data better than the null model (χ2 = 77.629, DF = 6, *P* < 0.001). However, a likelihood ratio test revealed that the three-way interaction of sex, species, and age was not significant (estimate = 0.299; SE = 0.158; *P* = 0.063). We ran a reduced model, excluding the three-way interaction, while including all two-way interactions (sex*species, age*sex, and age*species) and the random effects. In this reduced model none of the two-way interactions were significant (age*sex: estimate = − 0.014; SE = 0.078; *P* = 0.862; age*species: estimate = − 0.021; SE = 0.078; *P* = 0.791; species*sex: estimate = 0.280; SE = 0.159; *P* = 0.084). Further reducing the model revealed that the interactions of sex and age at sampling as well as species with age at sampling dropped out non-significant, while age at sampling remained as a main effect. This final reduced all-age-model revealed that with increasing age, α-Kl levels decreased significantly (Table 1 and Fig. 1). Older individuals (including α-Kl levels of individuals 20–49 years of age) showed a 2.5-fold decline in alpha klotho levels as compared to younger individuals (including α-Kl levels of individuals 1–9 years of age).

**Table 1:** Results of the final general linear mixed model obtained by analysing serum soluble alpha Klotho levels from chimpanzees and bonobos of both sexes, with species and sex in an interaction term, and age as a fixed effect

Term	Estimate	SE	DF	χ^2^	*P*-value
Intercept	6.802	0.071			
Sex	−0.046	0.116			
Species	−0.075	0.092			
Age at sample collection	− 0.367	0.042	1	27.452	**< 0.001**
Interaction sex with species	0.277	0.155	1	3.034	0.082

We correlated age at sampling time with α-Kl levels in chimpanzees and bonobos. A Spearman correlation of rank showed a significant negative correlation of α-Kl levels with age (*N* = 140, rho = − 0.632, *p* <  0.001).

### Soluble alpha klotho (α-Kl) levels in adult bonobos and chimpanzees

In a second LMM we investigated sex and species differences in α-Kl levels during adulthood (adults-only-model). In the adults-only-model, the full model was significantly different from the null model (χ2 = 24.946, DF = 7, *P* <  0.001), indicating that the test predictors age, sex, and species significantly influenced α-Kl levels in bonobos and chimpanzees older than 15 years. In this adults-only-model, the likelihood ratio test revealed that the three-way interaction of age, sex, and species was not significant (estimate = − 0.246; SE = 0.205; *P* = 0.187). We ran a reduced adults-only-model with all two-way interactions (sex*species, age*sex, and age*species) and random effects. As in the all-age-model, no two-way interaction including age at sampling was significant (age*sex: estimate = − 0.042; SE = 0.098; *P* = 0.666; age*species: estimate = 0.013; SE = 0.094; *P* = 0.892). However, the interaction of species and sex showed a significant effect on α-Kl levels (estimate = 0.642; SE = 0.186; *P* = 0.001), indicating that α-Kl levels differ between the two species and in a sex-specific way. Therefore, interactions with age at sampling day were removed, but the interaction of sex*species as well as age at sampling day as a main effect were kept in the second reduced adults-only-model. The results of the final reduced adults-only-model (including age at sampling and the sex*species interaction) revealed that also in adult individuals, α-Kl levels decline significantly with increasing age, and that males and females show significant differences in α-Kl patterns within a species (Table 2).

**Table 2:** Results of the final general linear mixed model obtained by analysing serum soluble alpha Klotho levels from adult chimpanzees and bonobos of both sexes, with species and sex in interactions, and age as a fixed effect

Term	Estimate	SE	DF	χ^2^	*P*-value
Intercept	6.637	0.086			
Sex	−0.283	0.139			
Species	−0.265	0.107			
Age at sample collection	−0.138	0.043	1	9.838	**0.002**
Interaction sex with species	0.659	0.178	1	12.369	**< 0.001**

**Fig. 2:**
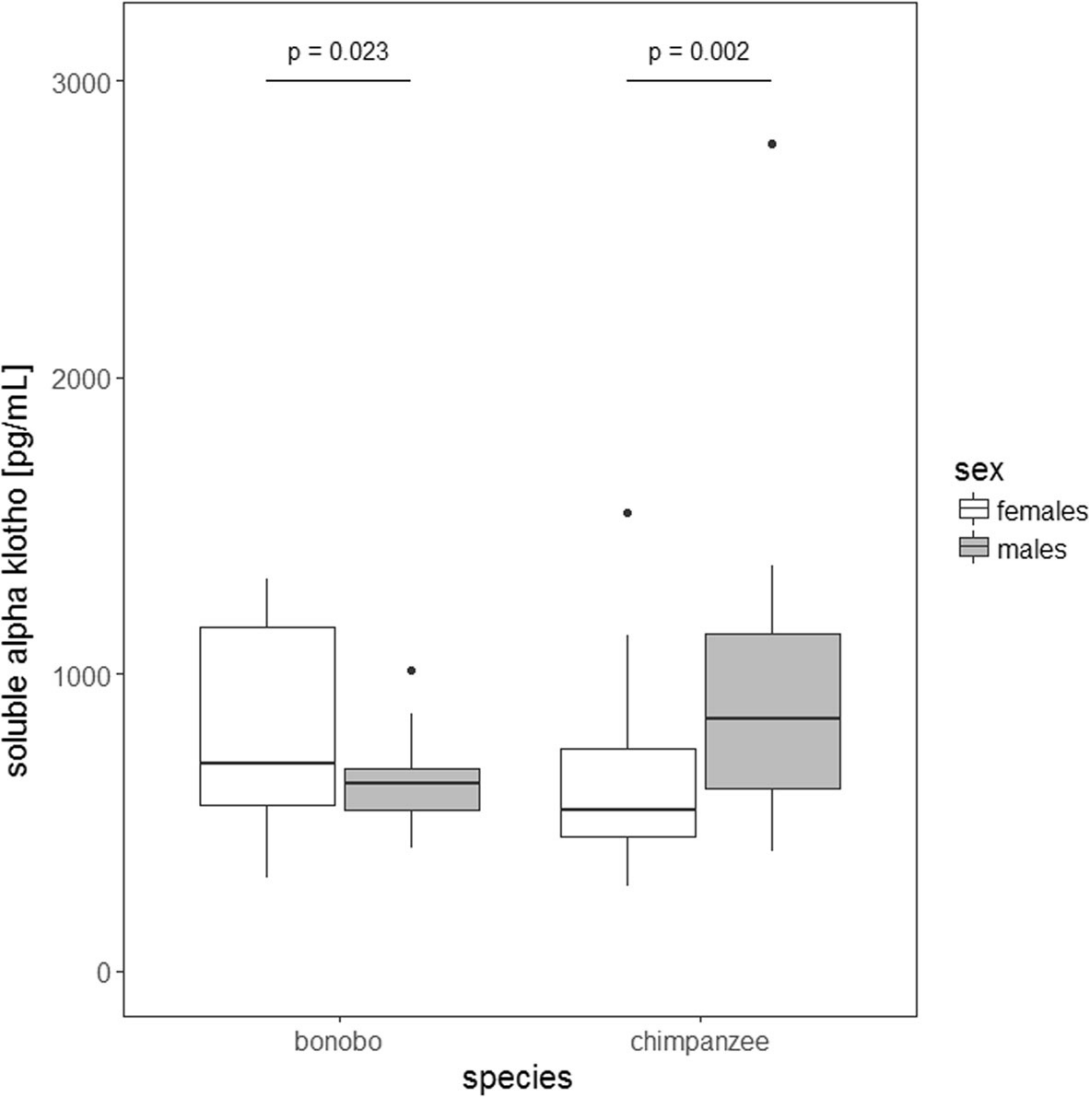
Average soluble alpha Klotho levels for adult female and adult male bonobos versus chimpanzees. The boxes illustrate the 25th and 75th percentiles, bars indicate medians, and circles indicate outliers. Sample sizes: N_bonobos_ = 31 (12 males, 19 females); N_chimpanzees_ = 52 (16 males, 36 females)

To further explore sex and species difference, we ran a post-hoc comparison and found that when controlling for age, male bonobos had significantly lower α-Kl levels compared to female bonobos (χ2 = 5.1794, DF = 1, *P* = 0.023) (Fig. 2). In chimpanzees, the opposite pattern was detected: females had significantly lower α-Kl levels than males (χ2 = 9.3714, DF = 1, *P* = 0.002) (Fig. 2).

## Discussion

Our study is the first to measure soluble α-Kl levels in two non-human primate species which, like humans, have long lifespans, slow rates of somatic growth, late onset of reproduction, and an extended period of adulthood. In chimpanzees and bonobos, soluble α-Kl levels significantly decreased from infancy to old age. As expected, we found differences in α-Kl levels between adult males and females, but unexpectedly the species had contrasting patterns of sex differences. In bonobos, females had significantly higher α-Kl levels than males, whereas in chimpanzees males had significantly higher α-Kl levels than females. If α-Kl levels reflect faster aging that is due to extrinsic factors such as intensity of intra-sexual competition [2], values of both species should be higher in females and the sex difference should be more pronounced in chimpanzees. While our measures of α-Kl in bonobo samples supported this hypothesis, those from chimpanzees did not. However, apart from this difference, the general pattern of decreasing α-Kl levels matches age-related changes of other hormones reported from humans and apes including thyroid hormones (T3) and IGFBP-3 [32, 67–70]. This suggests that age-associated changes in different physiological markers like T3 and IGFBP-3 should be assessed simultaneously in the two species. For example, human studies found that α-Kl levels are affected by the activity of other endocrine parameters. Increasing levels of arginine vasopressin (AVP) suppress α-Kl levels and vice versa [71, 72]. Bonobos and chimpanzees differ in vasopressin receptor gene expression with a deletion in that gene only occurring in chimpanzees [73]. Furthermore, males being homozygous in this deletion were more social than heterozygous males or males without deletion [74]. Thus, it would be informative to compare circulating AVP levels in the two species to see if this could explain the differences in α-Kl levels. Yet, to our knowledge, such data are not available.

As in humans, α-Kl levels of bonobos and chimpanzees decreased significantly with age [47, 75–77], but the negative correlation between α-Kl levels and age (ranging from *r* = − 0.16 [77] to *r* = − 0.255 [76]) in human samples was less pronounced than in our data set (*r* = − 0.632). One possible explanation is that our sample pool included individuals from all ages whereas measures obtained for human studies are restricted to specific age categories, namely either children [47], adults [76], or older adults [48]. Another explanation could be the shorter lifespan of chimpanzees and bonobos compared with humans. Given that the age-related decline of α-Kl levels in apes happens in a shorter time window than in humans, this could amplify the dynamics of hormone level changes.

Based on data from human studies [75, 76, 78], we had expected to find higher α-Kl levels for females than for males in both species. In humans, women and girls have higher α-Kl levels than men and boys, respectively [75, 76, 78]. Indeed we found this relationship in α-Kl levels for our samples from bonobos. The higher α-Kl levels in samples of adult female bonobos match their longer life expectancy in comparison to males. By contrast, results from chimpanzees deviate from our expectation as males had significantly higher α-Kl levels than females. These results do not support the hypothesis that sex-specific differences in α-Kl levels are influenced by male mate competition patterns in the two species. However, what we cannot exclude is that our results are influenced by differences in grouping patterns. Specifically, the chimpanzees involved in our study were often kept in one-male groups, thereby making aggressive male mating competition over mates obsolete. Future studies should compare the impact of demographic parameters such as group size and composition and, related variation in competition, with α-Kl levels.

One compelling question that our results raise is whether the differences in α-Kl levels between the sexes of the two *Pan* species are explained by sex-specific exposure to stressors in relation to their social system? Prather et al. [77] explored the association between exposure to long-term stressors and α-Kl levels in young women. One cohort consisted of women who were chronically stressed mothers of children with autism spectrum disorder, while the second group was mothers of healthy children. It was found that the cohort that was exposed to the stressor had lower α-Kl levels than women of the control group. Unfortunately, in this study, stress was assessed by self-reports [77]; cortisol or any other physiological measure of stress response was not collected. Extrapolating from a study that showed that caregivers of medically fragile children have elevated cortisol levels in comparison to control groups [79, 80], it is reasonable to assume that mothers exposed to the same chronic stressor in the study by Prather et al. [77] had also elevated cortisol levels. The physiological stress response seems to be directly or indirectly influenced by the pathways involving α-Kl, e.g., regulating insulin, fibroblast growth factor, and N-methyl-D-aspartate receptor signalling [77]. If the subordinate sexes in bonobos and chimpanzees face more stress exposure, this could explain variation in species differences in α-Kl levels. In bonobos, adult females are dominant over the majority of males and the highest ranking individual is always a female [81–83]. Furthermore, in captivity and in the wild female coalitions may charge males which is likely to induce elevated cortisol levels in males [84, 85]. In captivity, male bonobos have been shown to have higher salivary cortisol levels than females [86, 87]. Therefore, despite low levels of male mate competition, male bonobos face high levels of social stress, which may suppress α-Kl in individuals of the subordinate sex. Wild chimpanzees live in a male-dominated society in which females are subordinate to all adult males [88], and aggression from males towards females imposes physiological costs on females in terms of increased glucocorticoid secretion [89]. In addition to male aggression, higher glucocorticoid levels in wild female chimpanzees may also be due to limited access to food resources and low rank [90]. However, most of these stress-related factors like male competition and intergroup encounters are absent in captive male chimpanzees, still the life expectancy is shorter than in females. This shorter life expectancy of male chimpanzees corresponds with faster decline in IGFBP3-levels in male versus female chimpanzees [32], however, not in α-Kl levels. Therefore, in captive chimpanzee populations, neither difference in external factors nor in internal factors alone explains the sex differences in life expectancy. Among captive chimpanzees, the primary causes of morbidity and mortality are infectious diseases, disorders of the gastrointestinal and respiratory systems, and cardiac disease [91–96]. To what extent the observed differences in α-Kl levels between the sexes are reflected by genetic factors, diet, or interaction of other hormones like AVP, and how they align with behavioural and morphological indicators of development, remains to be investigated.

## Conclusion

In bonobos and chimpanzees, α-Kl levels decline with age suggesting that potential may arise from this marker for studying the process of aging on an individual, population, species, and on a comparative level. Bonobo females have higher α-Kl levels in comparison to males, which correspond to sex differences in life expectancy and mirrors sex differences found in humans. In chimpanzees, males have higher α-Kl levels than females posing a mismatch with sex differences in life expectancy. The observed sex differences α-Kl levels correspond with inter-sexual dominance relations of the two species and we cannot exclude that social stress and related cortisol levels affect α-Kl levels. Thus, considering interactions between α-Kl and other hormones, like glucocorticoids and AVP, potentially helps to explain species differences in α-Kl levels and how this relates to life expectancy and physiological aging.

## Methods

### Sample collection and subjects

We measured soluble α-Kl levels in 140 serum samples of 63 bonobos (42 females, 21 males) and 77 chimpanzees (52 females, 25 males) from 16 zoos (Table 3). Eight subjects, five bonobos and three chimpanzees, were sampled twice. After collection, samples were stored at − 20 °C in freezers of the veterinarian facilities of the respective zoos before they were shipped frozen to the Endocrinology Laboratory of the Max Planck Institute for Evolutionary Anthropology in Leipzig, Germany. All animals were kept in social groups. All bonobos lived in multi-male multi-female groups whereas many chimpanzees were kept in one-male multi-female groups. All apes had *ad libitum* access to fresh water, and food was offered at least three times a day and consisted mainly of a mixture of fruits and vegetables. All subjects had access to indoor and outdoor enclosures.

**Table 3:** Origin of serum samples used in this study

	bonobo		chimpanzee	
zoo	female	male	female	male
Apenheul	1	2	0	0
Basel	0	0	2	2
Berlin	4	1	0	0
Bremerhaven	0	0	2	1
Cologne	2	2	0	0
Frankfurt	2	2	0	0
Gossau	0	0	1	1
Leipzig	9	4	21	5
Lisbon	0	0	3	2
Magdeburg	0	0	1	3
Muenster	0	0	1	0
Munich	0	0	1	0
Planckendael	9	8	0	1
Stuttgart	6	0	10	2
Twycross	6	2	10	8
Wuppertal	3	0	0	0
sum	42	21	52	25

Our analyses included individuals aged from 1 to 48 years for bonobos (average age 17.5 years) and 3 to 49 years for chimpanzees (average age 22.9 years). For 54 (86%) of the 63 bonobos, the exact birthdates were known; exact birthdates were available for 63 (82%) of the chimpanzees. For one male and two female chimpanzees for whom only the year and month of birth were known, we set the day of birth to the 15th of the respective month. For nine bonobos (seven females and two males) and eleven chimpanzees (eight females and three males), exact age was not known and the day of birth was set optionally to June 15th of the estimated year of birth. Individuals for whom the age was estimated were mainly older and therefore the relative error of estimated age was relatively small.

For the analyses of α-Kl levels during adulthood, we created a subset with samples of individuals older than 15 years of age [97, 98]. This reduced data set contained 31 samples from bonobos (19 females, 12 males) and 52 samples from chimpanzees (36 females, 16 males) from 14 zoos.

### Soluble alpha klotho (α-Kl) measurement

We measured soluble α-Kl with a commercial ELISA (IBL International, REF JP27998). The assay was originally developed for the measurement of soluble α-Kl in human serum and EDTA (Ethylenediaminetetraacetic acid)-plasma samples. Before we used the ELISA for routine measurements, we tested for parallelism of diluted pool serum samples for each species separately. Pooled samples consisted of one female and one male sample of each species. Serially diluted pooled samples were found to parallel the standard curve (Additional file 1: Figure S1). Inter-assay coefficients of variation were 1.7% for high and 9.9% for low (*N* = 3), and the intra-assay coefficient of variation was 3.2%. Based on these results, the ELISA was considered to be appropriate for measuring α-Kl in serum samples of bonobos and chimpanzees. Two and more freezing cycles changed measured α-Kl levels by around 10% (details are presented in the supplementary material (Additional file 1: Figure S1)).

### Statistical analyses

We ran two linear mixed models (LMMs [66]), one to examine changes of α-Kl levels during life (all-age-model), and a second to investigate sex and species differences in α-Kl levels during adulthood (adults-only-model). Both models were run in R [99] using the function lmer provided in the package lme4 [100].

In the all-age-model investigating α-Kl levels during lifetime, the response variable, α-Kl, was log-transformed to approximate a normal distribution. The model included a three-way interaction of age (z-transformed), sex, and species (both dummy coded), as well as relevant random effects (see below). The three-way interaction was included in the all-age full model as mortality and life expectancy was expected to differ between sexes and species, and α-Kl is associated with human aging syndromes. We predicted that α-Kl levels decline with age in both species but at different times, or, depending on sex, with different steepness, leading to species-specific patterns that vary between the sexes. We included random intercepts for zoo-ID, to account for a possible influence of relevant animal husbandry conditions, and for subject, as eight individuals were included twice in the data set. There was considerable variation in terms of demography of ape groups across different zoo facilities; therefore, we included random slopes for age in zoo-ID.

Model assumptions (normal distribution and homogeneity of residuals) were assessed by visual inspections of a histogram, a q–q plot of the residuals, and by plotting residuals against fitted values. All model assumptions were met. We examined Variance Inflation Factors (VIF [101]) using the function vif of the R-package car [102] applied to a standard linear model excluding random effects. These indicated that collinearity was not an issue (maximum VIF: 1.087).

To investigate the significance of the predictors age, species, and sex and their interactions, we compared the full model with a null model including only the random effects using a likelihood ratio test ([103]; R function “anova”). Significance for all tests was set at *P* = 0.05.

For a comparison of α-Kl levels obtained from apes with results published for α-Kl levels in humans, we performed Spearman correlation of ranks, correlating age at sampling time with α-Kl levels in chimpanzees and bonobos.

The adults-only-model, investigating sex differences in adult individuals, was built in the same way as described for the all-age-model, but excluded data of individuals who were younger than 15 years of age at the time of sample collection [97, 98]. All model assumptions were tested and met, and maximum VIF was 1.063. For post-hoc comparisons we built two data subsets, one for bonobos and one for chimpanzees, with age at sampling as a control variable, random intercepts for zoos and subjects, and random slopes for age in zoo.

## Additional file

Additional file 1: Additonal assay valdiation steps. (DOCX 128 kb)

## Abbreviations

AVP: Arginine vasopressin
EDTA: Ethylenediaminetetraacetic acid
ELISA: Enzyme-linked Immunosorbent Assay
IGF-1: Insulin-like growth factor 1
IGFBP-3: Insulin-like growth factor binding protein 3
LMM: Linear mixed model
T3: Triiodthyronin, a thyroid hormone
VIF: Variance Inflation Factors
α-Kl: Soluble alpha klotho
