# Supplementary material for: Aging and sex affect soluble alpha klotho levels in bonobos and chimpanzees
Source: Front Zool. 2018 Sep 19;15:35. doi: 10.1186/s12983-018-0282-9 (PMC6146871; doi:10.1186/s12983-018-0282-9)
Supplement: Supplementary file 1 — Additonal assay valdiation steps. (DOCX 128 kb) [file 12983_2018_282_MOESM1_ESM.docx]

**Additional file 1**





Additional file 1: Figure S1. Serially diluted serum samples of bonobos and chimpanzees parallel the standard curve.

**Effects of freezing-thawing cycles on α-Kl levels**

To test for the effect of freezing-thawing cycles on α-Kl levels, we used one pooled bonobo sample containing samples from three males and three females and one pooled chimpanzee sample containing samples from four female and two males. In chimpanzees, after the first and second freeze-thaw cycles, a-Kl levels increased by 2 % and 10 %, respectively. In bonobos, after the first freeze-thaw cycle a 10 % increase in α-Kl was found and levels were stable thereafter.
